# The mechanical effects of CRT promoting autophagy via mitochondrial calcium uniporter down‐regulation and mitochondrial dynamics alteration

**DOI:** 10.1111/jcmm.14227

**Published:** 2019-04-02

**Authors:** Ziqing Yu, Xue Gong, Yong Yu, Minghui Li, Yixiu Liang, Shengmei Qin, Zibire Fulati, Nianwei Zhou, Xianhong Shu, Zhenning Nie, Shimo Dai, Xueying Chen, Jingfeng Wang, Ruizhen Chen, Yangang Su, Junbo Ge

**Affiliations:** ^1^ Department of Cardiology Shanghai Institute of Cardiovascular Diseases Zhongshan Hospital Fudan University Shanghai PR China; ^2^ Shanghai Institute of Medical Imaging Shanghai PR China; ^3^ Department of Cardiovascular Diseases Key Laboratory of Viral Heart Diseases Ministry of Public Health Shanghai Institute of Cardiovascular Diseases Zhongshan Hospital Fudan University Shanghai PR China; ^4^ Department of Echocardiography Shanghai Institute of Cardiovascular Diseases Zhongshan Hospital Fudan University Shanghai PR China

**Keywords:** autophagy, cardiac resynchronization therapy, heart failure, mitochondria, mitochondrial calcium uniporter

## Abstract

The mechanism of cardiac resynchronization therapy (CRT) remains unclear. In this study, mitochondria calcium uniporter (MCU), dynamin‐related protein‐1 (DNM1L/Drp1) and their relationship with autophagy in heart failure (HF) and CRT are investigated. Thirteen male beagle's dogs were divided into three groups (sham, HF, CRT). Animals received left bundle branch (LBB) ablation followed by either 8‐week rapid atrial pacing or 4‐week rapid atrial pacing and 4‐week biventricular pacing. Cardiac function was evaluated by echocardiography. Differentially expressed genes (DEGs) were detected by microarray analysis. General morphological changes, mitochondrial ultrastructure, autophagosomes and mitophagosomes were investigated. The cardiomyocyte stretching was adopted to imitate the mechanical effect of CRT. Cells were divided into three groups (control, angiotensin‐II and angiotensin‐II + stretching). MCU, DNM1L/Drp1 and autophagy markers were detected by western blots or immunofluorescence. In the present study, CRT could correct cardiac dysfunction, decrease cardiomyocyte's size, alleviate cardiac fibrosis, promote the formation of autophagosome and mitigate mitochondrial injury. CRT significantly influenced gene expression profile, especially down‐regulating MCU and up‐regulating DNM1L/Drp1. Cell stretching reversed the angiotensin‐II induced changes of MCU and DNM1L/Drp1 and partly restored autophagy. CRT's mechanical effects down‐regulated MCU, up‐regulated DNM1L/Drp1 and subsequently enhanced autophagy. Besides, the mechanical stretching prevented the angiotensin‐II‐induced cellular enlargement.

## 1 INTRODUCTION

Heart failure (HF) is associated with high risk of mortality and morbidity. The onset and progress of HF are closely related to mitochondria injury.^1^ The intact mitochondrial double‐layer membrane possesses the ionic selectivity, particularly, for calcium. Calcium‐overload in mitochondrial matrix leads to increased membrane permeability and dissipated membrane potential, and subsequent cell death is inevitable.^2^ The mitochondrial calcium uniporter (MCU) localized at the inner membrane of mitochondria confers the high selectivity of calcium. From the current perspective, MCU is the most important calcium channel mediating inward calcium flux into the mitochondrial matrix.^3^ Consequently, MCU determined mitochondrial calcium concentration and even buffered cytosolic calcium.^2^, ^4^, ^5^ The cytosolic calcium can influence the mitochondrial fission by the dynamin‐related protein 1 (DNM1L/Drp1),^5^ and such alteration of mitochondrial dynamics could further change the autophagy and mitophagy.^6^ It is reported that the level of autophagy is changed in HF, and the elevated autophagy is believed to be an important mechanism to degrade unnecessary cellular component. This process permits the sequential degradation and recycling of cellular components.^6^, ^7^ Autophagy can be influenced on condition of MCU function or expression anomaly.^8^, ^9^ Besides, impairment of autophagy in company with the suppressed DNM1L/Drp1 and mitochondrial fission in HF indicates that alteration of mitochondrial dynamics and autophagy should take part in the compensatory mechanism in HF.^6^ Additionally, variation of mitochondrial dynamics could further influence the mitophagy which is important to maintain cardiomyocyte integrity.^10^, ^11^, ^12^ In terms of HF treatment, recent years have witnessed great progression in cardiac devices development, especially the cardiac resynchronization therapy (CRT), an established biventricular‐pacing method to effectively improve cardiac function.^13^ CRT activates myocardium based on electromechanical coupling theory,^14^ and its mechanical effects are believed to correlate with cardiac function improvement.^15^ Although CRT shows impressive performance in clinical practice, the molecular mechanism of CRT still remains unknown. Considering a number of patients do not respond to CRT,^13^, ^16^ further study on the mechanism of CRT is helpful to deeply understand its principle and offer solution for improving the response rate of CRT. Consequently, distinct illumination of the mechanism of CRT on molecular level is important. In the present study, a canine model is introduced to investigate the CRT's effects on HF and its relationship with MCU and autophagy in vivo. To some extent, the ultimate effect of CRT on heart is the mechanical stretch of the ventricular wall. Therefore, a cell stretching culture method is applied to imitate CRT's mechanical effect on cardiomyocytes in vitro. In general, this study aims to disclose the important role of the alteration of MCU and DNM1L/Drp1 and their influences on autophagy in CRT. We suggest that decreased MCU, elevated DNM1L/Drp1 and subsequent enhanced autophagy after CRT provide cardioprotection. To our knowledge, the connection among MCU, DNM1L/Drp1 and autophagy to elucidate the molecular mechanism of CRT has not been reported before.

## 2 MATERIALS AND METHODS

### 2.1 Establishment of canine model with experimental heart failure

Thirteen male beagle's dogs with ages ranging from 1 year to 1.5 year and weights around 15‐20 kg were originally purchased from the College of Agriculture and Biology, Shanghai Jiaotong University for this study. Animals were randomly divided into three groups: sham group (n = 4), HF group (n = 5), CRT group (n = 4). However, as one dog in HF group died from serious post‐operation infection, 12 dogs were finally included. The asynchronous HF was established through left bundle branch (LBB) ablation followed by 8 weeks of rapid atrial pacing with 200 bpm. The LBB potential was mapped and characterized as biphasic or triphasic waves between His bundle potential and ventricular potential. LBB block was verified by intracavitary electrocardiograph after frequency ablation. CRT group was treated with 4 weeks of rapid atrial pacing followed by 4 weeks of biventricular pacing at the same heart rate (Figure S1). At terminal study, dogs were anaesthetized and samples were collected as previously described 50‐100 mg aliquots from the lateral walls of each dog's left ventricle were used for experiments. Protocols were complied with the published Guide for the Use and Care of Laboratory Animals published by the National Institutes of Health.

### 2.2 Echocardiography

Echocardiography was performed at three time points (baseline, 4‐6 weeks after HF model being set up and 8 weeks after CRT operating) by the same experienced cardiologist who was blind to the grouping of animals, using a Philips IE33 instrument (Philips Medical Systems Corporation, Andover, MA, USA) with a 1‐5 MHz transducer (S5‐1), under the instruction of the guideline.^17^ Left ventricular ejection fraction (LVEF) was detected by Simpson's method with parameters of left ventricular end systolic volume (LVESV) and left ventricular end diastolic volume (LVEDV). Color M‐mode tissue Doppler imaging (TDI) from parasternal long‐axis view and apical four‐chamber view was used to evaluate the septal‐to‐posterior wall motion delay (SPWMD), a screening method of left ventricular asynchrony defined as the delay between maximum displacement of the septum and of the posterior wall.

### 2.3 Electrocardiography

Electrocardiographs (ECGs) were performed to validate the LBBB and biventricular pacing. All ECGs were depicted at a paper speed of 25 mm/s. QRS duration and QRS morphology were measured by two independent cardiologists. When their opinions went against each other, another physician was brought in to give an ultimate decision.

### 2.4 Microarray analysis of differentially expressed genes

Tissue samples of lateral LV wall in the distribution of the left circumflex artery were acquired and mixed from four dogs in each group, and the total RNA was isolated with TRIzol reagent (Invitrogen Life Technologies, Carlsbad, CA) after homogenization (Figure S4A). Four pieces of mixed RNA were quantified by the spectrophotometers (NanoDrop ND‐1000, Thermo Scientific Inc, Wilmington, DE) respectively. Additionally, RNA integrity was assessed by standard denaturing agarose gel electrophoresis. Afterwards, total RNA was amplified and labelled with the dye Cy3. Then the labelled RNA was hybridized onto the canine specific Whole Genome Oligo Array (4x44K, Agilent Technologies Inc, Palo Alto, CA, USA). After having washed the slides, the arrays were scanned by the microarray scanner (G2505C, Agilent Technologies Inc, Palo Alto, CA, USA). Data were extracted by Agilent Feature Extraction software (version 11.0.1.1), and subsequent data processing were performed with the GeneSpring GX v12.1 software package. After the quantile normalization of raw data, genes that at least one of three samples have flags in Detected (“All Targets Value”) were chosen for further data analysis. Genes with more than 4‐fold changes were identified as differentially expressed ones, and hierarchical clustering analysis was performed to show the distinguishable gene expression pattern among three independent mixed samples. Finally, gene ontology (GO) and protein‐protein interaction (PPI) analysis were applied through the STRING online tool,^18^ Cytoscope software^19^ and DAVID.^20^ Fisher's exact test is used to find if there is more overlap between the DEGs and the GO terms that would be expected by chance, and *P *< 0.05 was considered statistically significant.

### 2.5 Morphological examination

The hearts of the murine models were promptly obtained, and then fixed in formalin, embedded in paraffin, sliced into 5‐μm thick sections, and stained by Haematoxylin & Eosin (H&E) and Masson's trichrome, with the use of standard histology techniques. From the histological analysis, the cross‐sectional area (CSA) through H&E stain and the fibrotic area percentage (FAP) through Masson's trichrome stain were investigated. A transmission electron microscope (TEM) was performed to disclose the morphological changes of mitochondria, autophagosomes and mitophagosomes. Cardiac tissue was fixed in 2.5% glutaraldehyde for more than 24 hours, dehydrated by gradient ethanol, embedded with pure acetone, solidified in oven, sliced and stained with gold. TEM image was finally acquired by an electron‐microscopic system (FEI Tecnai G2 Spirit).

### 2.6 Cell stretching culture and validation of the effect of MCU inhibitor

To date, it was hardly seen the simulation of CRT on the cellular level. In the current viewpoints, CRT improved cardiac function and asynchrony through promoting the mechanical function of delayed myocardium.^21^ Even baseline myocardial mechanical stretching could be regarded as a predictor of CRT response and good prognosis.^22^ It's reasonable to deduce that CRT ultimately influenced the heart by stretching the cardiomyocyte. Therefore, the cardiomyocyte stretching culture was introduced to imitate the mechanical effect of CRT (Figure S7A). Cells were divided into three groups: control group, angiotensin‐II group and angiotensin‐II+stretching group. The murine ventricles were harvested from neonatal Sprague‐Dawley rats within 48 hours of birth. Then they were minced into pieces and digested in the tyrisin (0.1%). After the digestion, the cardiomyocyte suspension was centrifuged at 1000 × g for 5 minutes at 4°C, and the pellet was re‐suspended in low‐glucose DMEM culture (Gibco) containing 10% foetal bovine serum (Gemini). Afterwards, cardiomyocytes were purified by the differential adhesion method. The suspended cardiomyocytes were collected and seeded in a flexible silicone chamber consisting of 400‐μm thick outer walls and a 200‐μm thick membrane bottom (Strex Chambers, B‐Bridge International, CA, USA). The primary cardiomyocytes were divided into three groups: control group, angiotensin‐II group and angiotensin‐II+stretching group. After the cardiomyocytes adhering to the silicone membrane, the pathological condition was induced by 1 × 10^−6 ^mol/L angiotensin‐II in both angiotensin‐II group and angiotensin‐II+stretching group for 72 hours, while the control group was treated with PBS. In the angiotensin‐II+stretching group, a 10% increase in the length of the silicone chamber was achieved by fixing it on a chamber stand to impose mechanical stretch on cardiomyocytes for 36 hours (Figure S7B). Additionally, in vitro validation of the effect of MCU on autophagy/mitophagy was carried out by MCU inhibitor Ru360 at neonatal rat primary cardiomyocytes. Cells were acquired as abovementioned, treated with Ru360 (50 nmol/L), and incubated for 48 hours. Besides, the effects of a small interfering RNA (siRNA) mediated MCU knockdown in Ang‐II treated H9c2 cells was exhibited (see supplemental method for more detail).

### 2.7 Immunofluorescence

After cell stretching, the silicone membrane loaded with cardiomyocytes was fixed in 4% paraformaldehyde, cut into tiny pieces and blocked by the donkey serum. Cardiomyocytes were incubated with primary antibodies of MCU (1:50, orb317655, biorbyt), DNM1L/Drp1 (1:100, D6C7, CST) and GJA1/connexin43 (1:200, ab11369, Abcam), respectively. GJA1/connexin43 was used to show the localization and the contour of the cardiomyocyte. Moreover, the contour of the cardiomyocyte indicated by GJA1/connexin43 was used to assess the cellular cross‐sectional area (CSA). After incubation of fluorescent secondary antibodies (Invitrogen Alexa Fluor 488 and 555) and nucleophilic dye 4′,6‐diamidino‐2‐phenylindole (DAPI), the expression of MCU, DNM1L/Drp1 and GJA1/connexin43 in cardiomyocytes inoculated on the silicone membrane was detected by confocal laser scanning microscope (Leica TCS SP8).

### 2.8 Western blot

Tissue of lateral LV wall was homogenized in RIPA lysis buffer (Beyotime, Nantong, China) with protease inhibitor cocktail (Sigma Chemical Co, St. Louis, MO, USA). Proteins were acquired by ultracentrifugation, determined by BCA method, separated on a 12% SDS‐PAGE and transferred to polyvinylidene difluoride membranes. After dilution and blocking, the membranes were then incubated with primary antibodies of MCU (1:800, orb317655, biorbyt), DNM1L/Drp1 (1:1000, D6C7, CST), LC3B (1:1000, 2775, CST), SQSTM1/p62 (1:1000, 5114, CST), Parkin(1:1000, 4211, CST), Pink(1:1000, ab23707, Abcam) and β‐actin (1:10000, KC‐5A08, Kangcheng) at 4°C overnight. Incubated with rabbit‐radish peroxidase‐conjugated secondary antibody, the binding reactions in the membranes were detected by enhanced chemiluminescence assays.

### 2.9 Statistical analysis

Continuous variables were presented as mean ± standard error of mean (SEM) with at least three repeated experiments, and inter‐group difference was assessed by Student's *t* test or ANOVA via SPSS software 19.0. A *P *< 0.05 was regarded as significant.

## 3 RESULTS

### 3.1 Assessment of canine model and the effects of CRT in vivo

**Figure 1:**
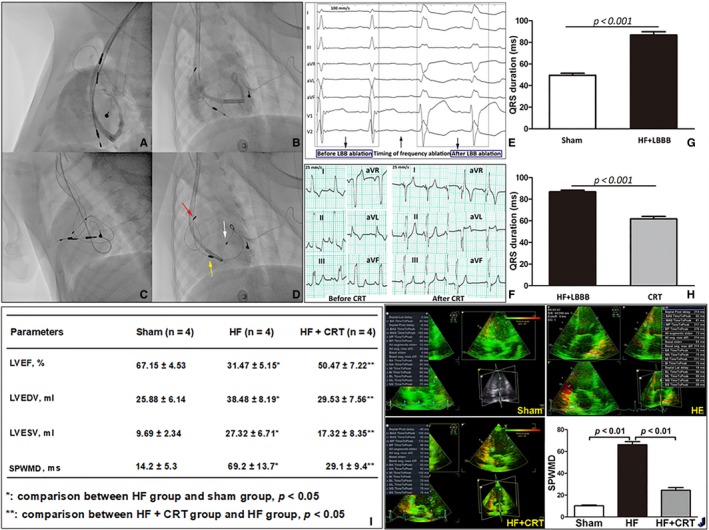
The validation of canine model and the evaluation the effect of cardiac resynchronization therapy

Body weight, echocardiographic parameters and QRS duration at baseline showed insignificant difference among three groups (Figure S2). The LBB potential was mapped and characterized as biphasic or triphasic waves between His bundle potential and ventricular potential. LBBB was verified by intracavitary electrocardiograph after frequency ablation (Figure 1E). Overdrive pacing‐induced heart failure was confirmed by decrease in LVEF and myocardial asynchrony. Compared with sham group, LVEF outstandingly decreased in HF group 65.78 ± 3.26 vs 40.47 ± 1.34, *P *<* *0.05) after 4 weeks of overdrive pacing (Supplemental table). After LBB ablation, the QRS duration was prolonged (86.8 ± 3.1 vs 49.5 ± 1.9 ms, *P *<* *0.001), while it was narrowed after CRT initiation (Figure 1G). After 4 weeks of CRT treatment, LVEF, LVESV, LVEDV and SPWMD were markedly improved in HF+CRT group compared with HF group (Figure 1I).

### 3.2 CRT decreasing cardiomyocyte's size, alleviating cardiac fibrosis, promoting the formation of autophagosome and mitophagosome and mitigating mitochondrial injury

In HF group, the CSA and the FAP increased dramatically compared with sham group, while CRT could alleviate the cell enlargement and myocardial fibrosis (Figure 3A,B). TEM study revealed an eminent increase of autophagosome, mitophagosome and mitochondrial fission in CRT group (Figure 3C). Moreover, TEM study indicated severe swelling and disruption of myocardial fibre in HF group (Figure S3B), while CRT could relieve these damages (Figure S3C). Besides, morphological disorder of mitochondria (disarrangement and cristae loss) indicated mitochondrial damage in HF dogs. However, after CRT treatment, the mitochondrial injury was significantly relieved (Figure 3C and Figure S3).

### 3.3 CRT significantly influencing gene expression profile

**Figure 2:**
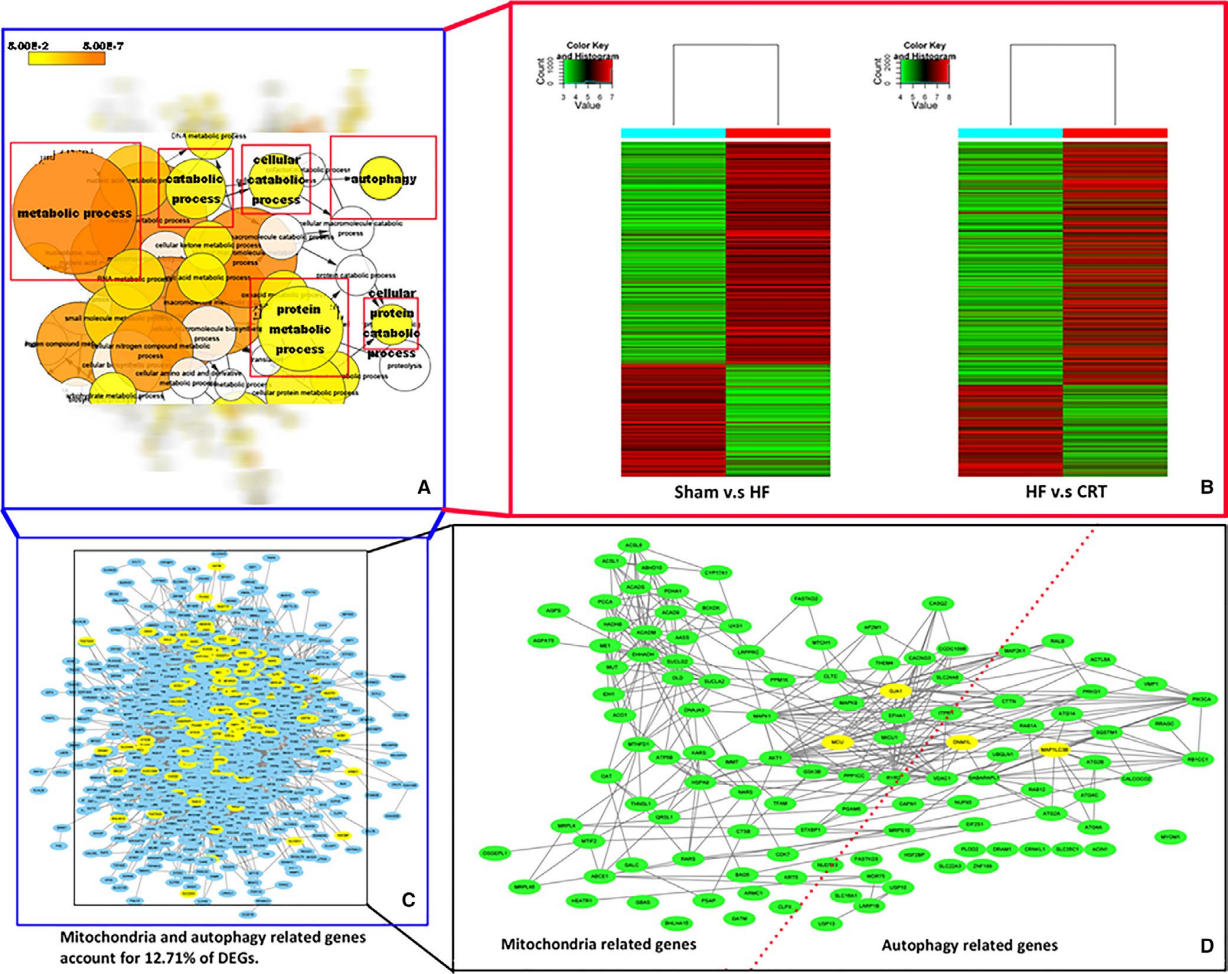
Analysis of differentially expressed genes

GO analysis of DEGs indicated kinds of biological processes involved (Figure 2A), especially various mitochondria‐associated processes (*P < *0.05) and autophagy (*P < *0.001). Meanwhile, gene cluster analysis validated the DEGs abounded with plenty of genes related to metabolism and autophagy (Figure 2B and Figure S6). Further analysis revealed the proportion of mitochondria‐ and autophagy‐related genes in the total DEGs was 12.7% (Figure 2C). The PPI analysis showed dense nodes with close PPI relationships among mitochondrial proteins and autophagic proteins (Figure 2D), and the densely linked proteins, including MCU, DNM1L/Drp1, Microtubule‐Associated Protein‐1 Light Chain‐3B (LC3B or MAP1LC3B), Sequestosome‐1 (SQSTM1/P62), Connexin43/GJA1 were verified in the subsequent experiment.

### 3.4 CRT down‐regulating the expression of MCU and up‐regulating DNM1L/Drp1 in vivo

**Figure 3:**
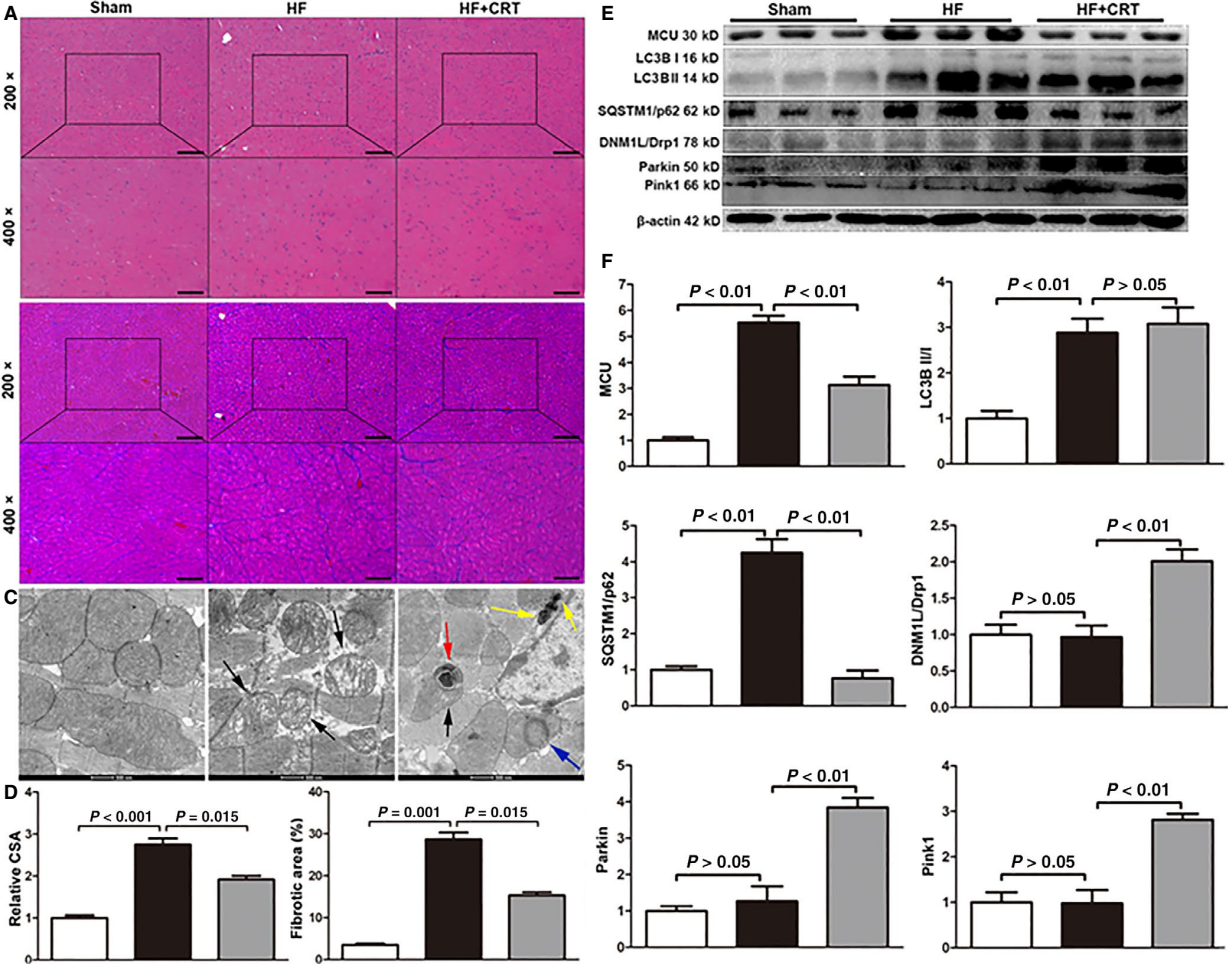
In vivo analysis of the pathological changes and the protein expression changes

Compared with sham group, MCU was up‐regulated in HF group (*P *<* *0.01). However, CRT could notably decrease MCU expression This change of MCU expression in HF and CRT treatment hinted that MCU should be a potential target of CRT. The expression of DNM1l/Drp1 showed no difference between sham group and HF group, however, it was elevated in HF+CRT group. The LC3B II/I ratio was elevated in HF group and HF+CRT group. However, the level of p62 was higher in HF group than the one in sham group and HF+CRT group. On one hand, elevated LC3B II/I ratio indicated autophagosome formation, and on the other hand, down‐regulation of p62 signified autophagolysosome formation, namely fluent autophagic flux (Figure 3E,F). This phenomenon suggested a blocked autophagic flux in failing heart. Nonetheless, CRT strengthened both autophagosome formation and autophagic flux in heart failure. In accordance with the TEM study, Pink1 was down‐regulated in HF group, while both Parkin and Pink1 were up‐regulated in HF+CRT group, indicating enhanced mitophagy (Figure 3E,F).

### 3.5 Effects of cardiomyocyte stretching culture on angiotensin‐II‐induced pathological stress

**Figure 4:**
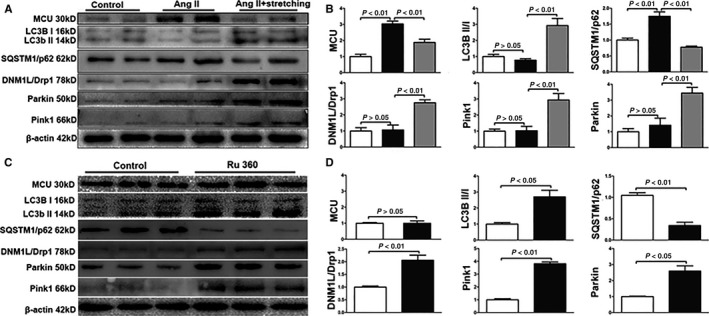
Analysis of the protein expression changes after cell stretching or mitochondria calcium uniporter inhibition in vitro

**Figure 5:**
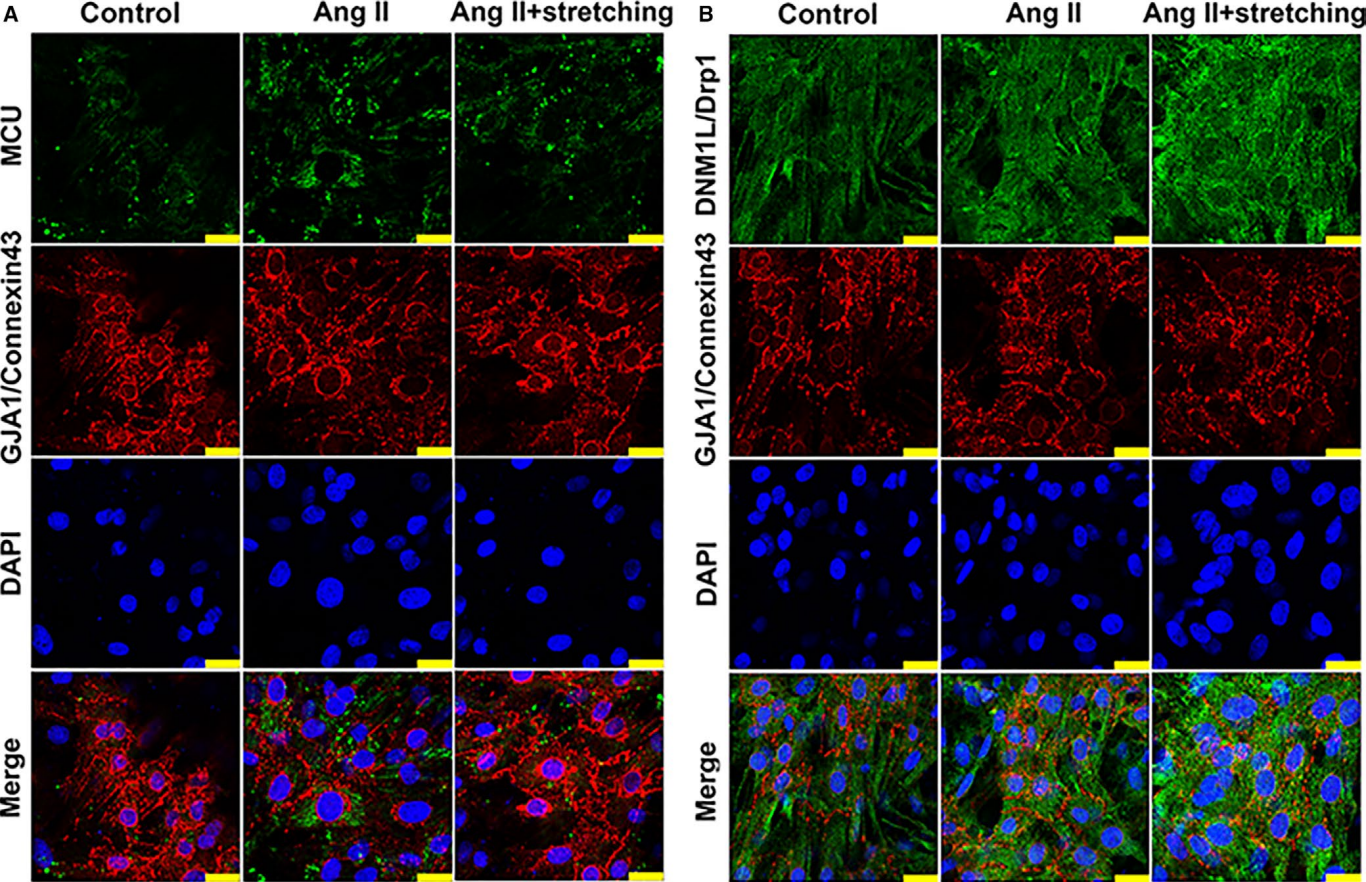
In vitro analysis of the effects of cellular mechanical stretching on angiotensin‐II treated cardiomyocytes

Consistent with the results of animal model, MCU was elevated in angiotensin‐II treated cells compared with control cells, however, cell stretching could reduce the expression of MCU and increase the expression of DNM1L/Drp1 under angiotensin‐II‐induced pathological stress (Figure 4A,B). LC3B II/I ratio was reduced, while SQSTM1/p62 was increased after angiotensin‐II treatment, indicating impaired autophagy. However, cell stretching could up‐regulate LC3B II/I, Parkin and Pink1 and down‐regulate SQSTM1/p62, referring to enhancement of autophagy and mitophagy (Figure 4A,B). Changes of intracellular fluorescence intensity of MCU and DNM1L/Drp1 were consistent with the results of western blots (Figure 5). Immunofluorescence of the cardiomyocyte membrane‐specific GJA1/connexin43 labelled cells showed that the CSA in angiotensin‐II group increased, while the cellular mechanical stretching could reverse angiotensin‐II‐induced cell enlargement (Figure S8). Ru360 treated primary cardiomyocytes showed enhanced autophagy and mitophagy in comparison with control cells (Figure 4C,D), indicating MCU inhibition directly elevating autophagy and mitophagy in vitro. To consolidate the concept that interference on MCU influencing cardiac autophagy in H9c2 cells incubated with Ang‐II, an siRNA sequence was designed (GCTGACTGCCGGCTGCTTTCC) to knockdown MCU. Ang‐II‐induced cell injury showed impaired autophagy and mitophagy in control cells, while MCU knockout could partly restore them. Therefore, intervention on MCU could protect from pathological injury by autophagy/mitophagy enhancement (Figure S9).

## 4 DISCUSSION

CRT brings great benefits to HF patients, especially to someone with refractory HF which hardly response to medication therapy. CRT was proved to improve cardiac function,^14^, ^21^, ^22^, ^23^, ^24^, ^25^, ^26^, ^27^, ^28^, ^29^ and our previous research indicated CRT alleviated cardiac fibrosis.^30^ Nonetheless, the mechanism of CRT remains unclear to date. From this study, CRT could increase LVEF, decrease LVESV, shorten QRS duration, reduce the size of cardiomyocyte, alleviate myocardial fibrosis and improve ventricular synchronous motion. The present study firstly disclosed that CRT could down‐regulate MCU and up‐regulate DNM1L/Drp1. Furthermore, autophagy and mitophagy were subsequently influenced in CRT‐treated HF animal model. In addition, to investigate the mechanical effects of CRT in vitro, an angiotensin‐II‐induced pathological stress and cell stretching model were adopted. Angiotensin‐II stimulation could elevate MCU, lower DNM1L/Drp1 and impair autophagy and mitophagy, while cell stretching could reverse these effects. Besides, the mechanical stretching could positively prevent the angiotensin‐II‐induced cellular enlargement. Ultimately, MCU inhibitor Ru360 could directly promote autophagy and mitophagy in vitro. LC3B II/I ratio was increased in in vivo study, however, this ratio was decreased in in vitro study. It was suggested that LC3B should be accumulated if the combination of autophagosome and lysosome is interrupted.^31^ In this condition, although autophagy was impaired, LC3B II/I ratio could be elevated. On the contrary, in spite of decreased autophagy triggered by persistent angiotensin‐II treatment,^32^ angiotensin‐II‐induced pathological condition for 72 hours might not be long enough to cause LC3B accumulation at all.

Barth et al firstly reported a microarray analysis of DEGs and related pathways on CRT‐treated HF dogs. They found CRT could significantly influence the metabolic pathway, however, the throughput of the microarray was not high enough.^24^ Another animal study showed CRT could improve HF by changing the energetic metabolism and increasing the level of ATP.^29^ Besides, CRT could improve the systolic function by regulating the activity of glycogen synthase kinase‐3β to influence the phosphorylation of excitation‐contraction coupling‐related proteins.^23^ DeMazumder et al. found CRT could re‐balance the sympathetic‐parasympathetic system through inhibiting the cholinergic pathway and improve cardiac function.^27^ It was reported that CRT could partly correct the heterogeneously expressed ryanodine receptor and sodium channel in HF to mitigate cardiac dysfunction and myocardial asynchrony.^25^, ^26^ CRT had effect on the oxidative modification of cysteine residue in ATP synthase, and the function of ATP synthase was regulated subsequently to increase the ATP production.^33^ Recently, a translational study reported microRNA‐30d could effectively predict CRT non‐response in patients, and their animal experiment suggested elevated circulating microRNA‐30d and cardiac in situ expressed microRNA‐30d might be the key factor against apoptosis.^28^ Abovementioned studies suggested CRT should influence cardiomyocyte on energetic metabolism. Therefore, roles of autophagy and mitophagy in CRT were investigated herein. To date, it was hardly seen the simulation of CRT on the cellular level. CRT improved cardiac function and asynchrony through promoting the mechanical function of the heart.^14^, ^21^ Moreover, baseline myocardial mechanical stretching could be regarded as a prognostic predictor of CRT response.^22^ It is reasonable to deduce that CRT ultimately influenced the heart by stretching the cardiomyocyte. Mechanical cell stretching promoted the metabolic activity of ventricular cells and optimized the utilization of ATP production.^34^

Our findings indicated increased MCU, decreased DNM1L/Drp1 and impaired autophagy and mitophagy in HF. MCU was believed to take an active part in mitochondrial pathophysiology.^2^ Zaglia et al reported MCU was elevated in angiotensin‐II‐induced cardiac hypertrophy, while microRNA‐1 could inhibit MCU's expression and alleviate cardiac dysfunction.^35^ MCU knockout could promote autophagy in vivo or in vitro,^8^, ^36^ and our previous study verified up‐regulation of autophagy and mitophagy after MCU knockdown in H9c2 cells originating from mouse cardiomyocytes.^9^ DNM1L/Drp1 was closely related to mitochondrial fission and mitophagy. In decompensated HF, autophagy and mitophagy were suppressed, coinciding with down‐regulated mitochondrial translocation of DNM1L/Drp1.^6^ MCU silencing could promote DNM1L/Drp1‐mediated mitochondrial fission and apoptotic cell clearance, avoiding the pathologic accumulation of metabolic products in vivo.^5^ Based on these thought‐provoking studies, we reckon that insufficiency of autophagy and mitophagy was underlying the initiation and maintenance of HF, while CRT could change the expression of MCU and DNM1L/Drp1 and partly restore cardiac autophagy.

### 4.1 Limitation

The Beagle's dogs in this study were of wild type, and MCU gene loss‐of‐function study could not be carried out as the transgenic dog model was unfeasible. Besides, the animal sample size was relatively small, and this study should be treated as a pilot study which needs more research in future. The cell stretching model was designed to imitate the mechanical effects of the CRT, however, the CRT‐related electrical effects could not be elucidated by the present study. Therefore, it is worthy of further investigation of the mechanism of CRT.

## 5 CONCLUSION

The mechanical effects of CRT could down‐regulate MCU, up‐regulate DNM1L/Drp1 and subsequently enhance autophagy and mitophagy. Persistent angiotensin‐II stimulation could elevate MCU and impair autophagy, while cell stretching could reverse these effects by decreasing MCU and increasing DNM1L/Drp1. Besides, the mechanical stretching could positively prevent the angiotensin‐II‐induced cellular enlargement. Inhibiting or knocking down MCU could promote autophagy and mitophagy in vitro. Based on in vivo and in vitro studies, the mechanical effects of CRT promoted autophagy via MCU down‐regulation and DNM1L/Drp1 up‐regulation.

## CONFLICTS OF INTEREST

None.
