# Supplementary material for: The mechanical effects of CRT promoting autophagy via mitochondrial calcium uniporter down‐regulation and mitochondrial dynamics alteration
Source: J Cell Mol Med. 2019 Apr 2;23(6):3833–42. doi: 10.1111/jcmm.14227 (PMC6533471; doi:10.1111/jcmm.14227)
Supplement: Supplementary file 1 [file JCMM-23-3833-s001.docx]

**Supplemental Materials**

**Supplemental method**

**Small interfering RNA (siRNA)**

The rat cardiomyocyte-originated H9c2 cell was treated with Ang-II and transfected with siRNA plasmid for 72 hour. The interfering sequence (GCTGACTGCCGGCTGCTTTCC), connecting with a green fluorescent protein (GFP) gene was integrated and amplified in Escherichia coli bacteria system (Clontech). H9c2 cells were transfected with plasmid through lipofectamine 2000 transfection kit (Thermofisher Scientific). Successful transfection was verified by GFP under fluorescence microscope investigation, and MCU knock-down was verified by WB-analysis.

**Supplemental table. Echocardiographic parameters difference between the sham group and the HF group after 4 weeks of overdrive pacing**

| **Echocardiographic parameters** | **Sham group (n=4)** | **HF group (n=8)** |
| --- | --- | --- |
| LVEF, % | 65.78±3.26 | 40.47±1.34* |
| LVEDV, ml | 26.61±4.10 | 33.48±7.57* |
| LVESV, ml | 9.73±1.98 | 19.32±4.66* |
| SPWMD, ms | 15.8±4.2 | 57.5±12.8* |

*: the comparison between the sham group and the HF group after 4 weeks of overdrive pacing, *p*< 0.05

**Supplemental figure 1.** The schematic overview of the overdrive pacing and biventricular pacing protocol


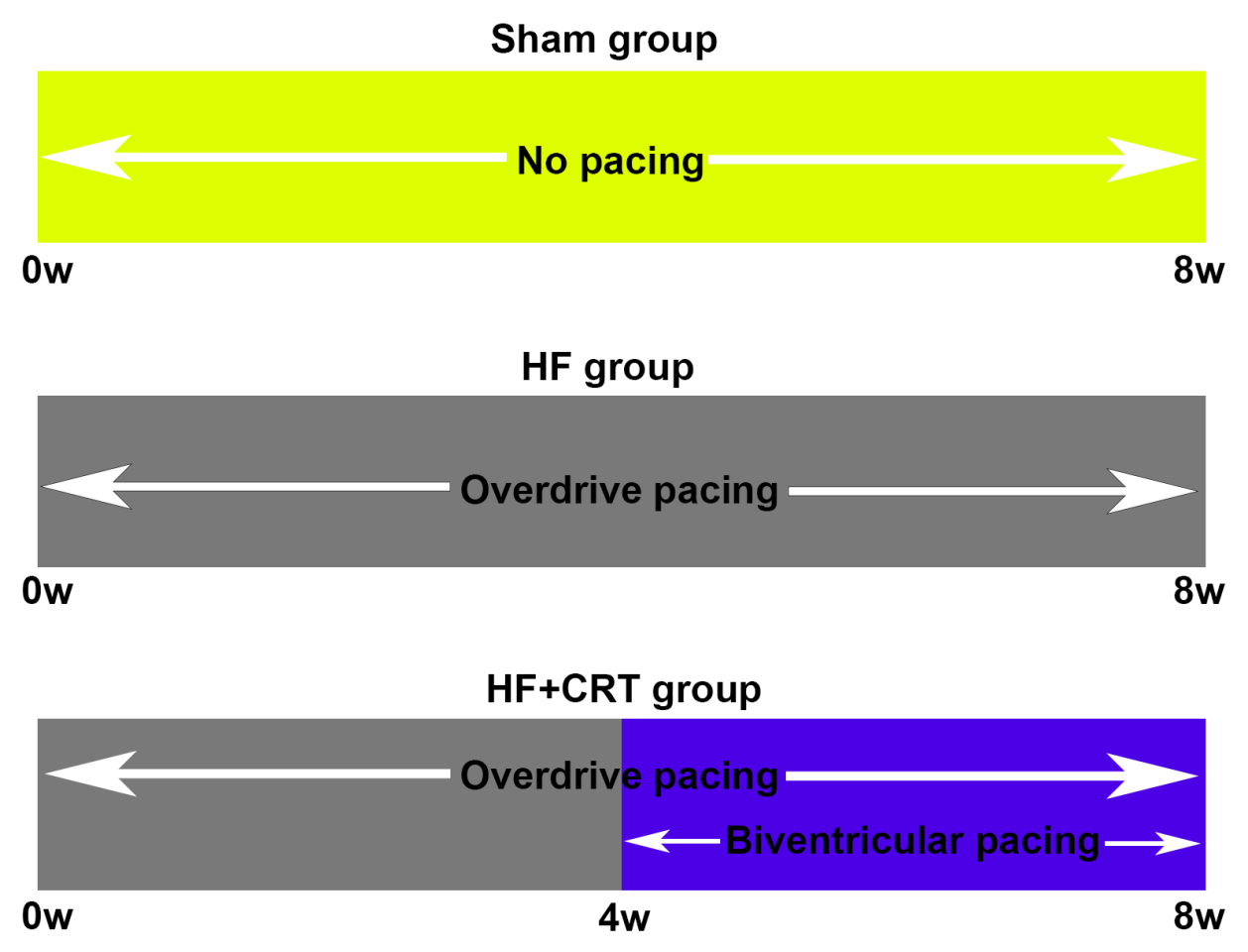


**Supplemental figure 2. No difference of body weight, echocardiographic parameters, and QRS duration at baseline among sham group, HF group, and HF + CRT group**


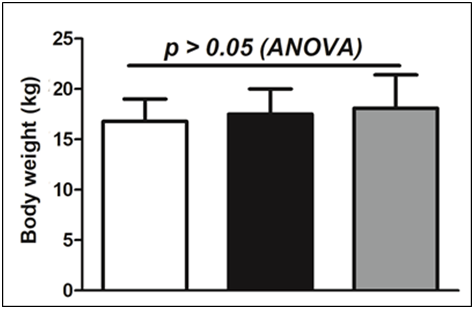


White column refers to sham group; black column refers HF group; grey column refers to HF + CRT group.

**Supplemental figure 3. Difference ofthe percentage of the injured mitochondria**


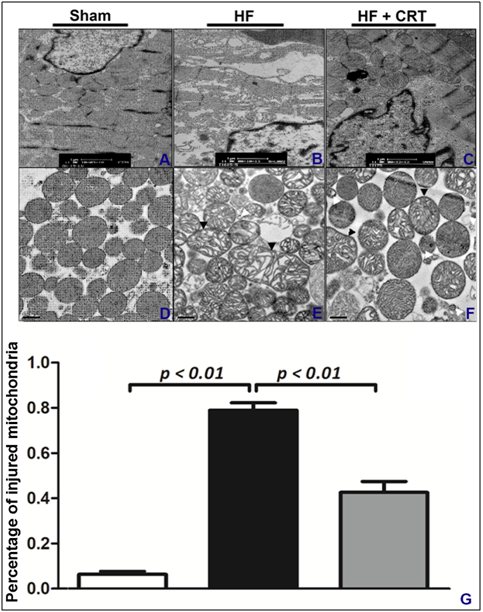


In sham group, the mitochondria from the lateral left ventricular wall remain compact and normal size, and the mitochondrial cristae appear dense. In the HF group, the mitochondria from the lateral left ventricular wall are swollen, and the mitochondrial cristae are sparse. In the HF + CRT group, the size of the mitochondria are smaller compared with the HF group, while the degree of mitochondria injury is significantly mitigated. The scale bar in A~C refers to 1μm, while the scale bar in D~F refers to 0.5μm.White column refers to sham group; black column refers HF group; grey column refers to HF + CRT group.

**Supplemental figure 4. Sample mixing protocol and the box plot to show the normalization of the raw data**

**
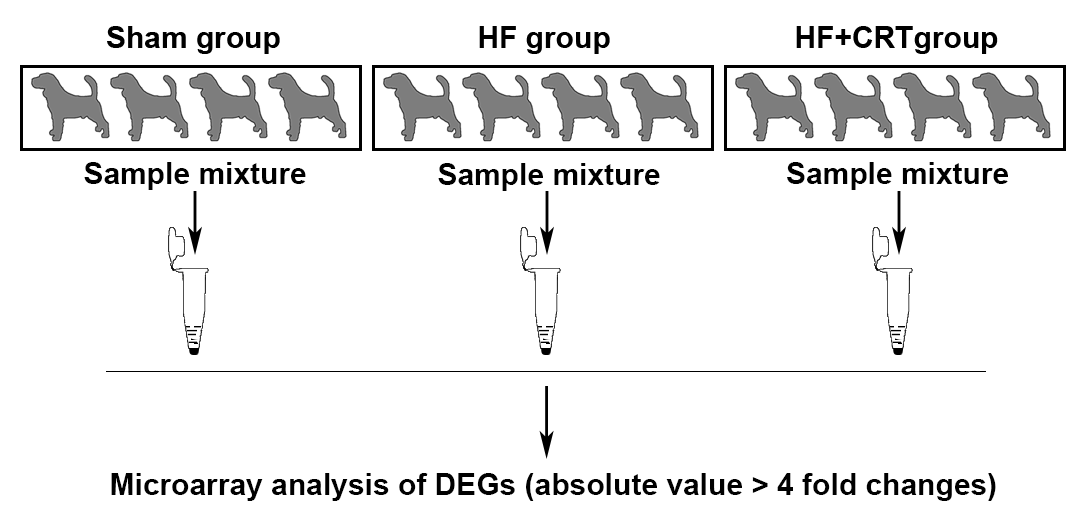
**

**A**


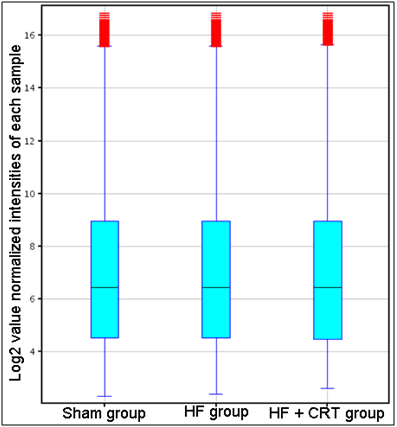


**B**

A: Sample mixing protocol and the threshold of the DEGs inclusion

B: The Box Plot is a convenient way to quickly visualize the distributions of a dataset. It is commonly used for comparing the distributions of the intensities from all samples. After normalization, the distributions of log2-ratios among the samples are nearly the same.

**Supplemental figure 5. The scatter plot to show the differentially expressed genes**


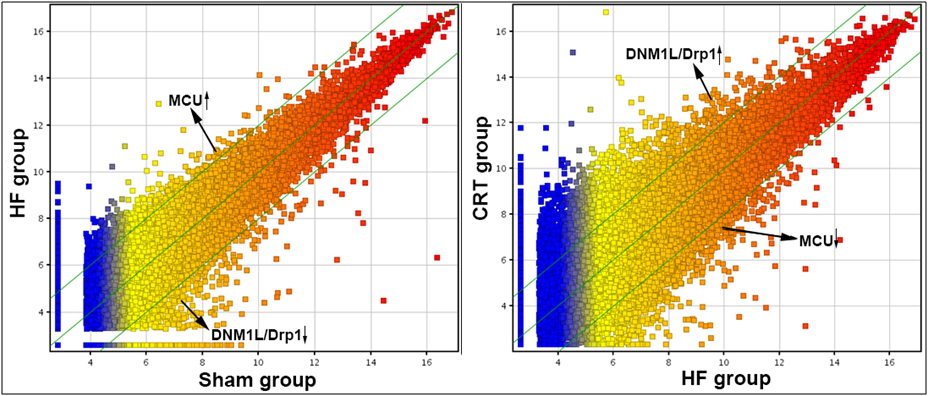


The Scatter-Plot is a visualization method used for assessing the gene expression variation (or reproducibility) between arrays. The values of X and Y axes in the Scatter-Plot are the normalized signal values of the compared samples or averaged normalized signal values of the compared groups. The green lines are Fold Change Lines (The default fold change value given is 4.0). The genes above the top green line and below the bottom green line indicated more than 4.0 fold change of genes between two samples or groups. MCU: mitochondria calcium uniporter; DNM1L/Drp1: dynamic related protein-1.

**Supplemental figure 6. Venn analysis and GO analysis of differentially expressed genes**


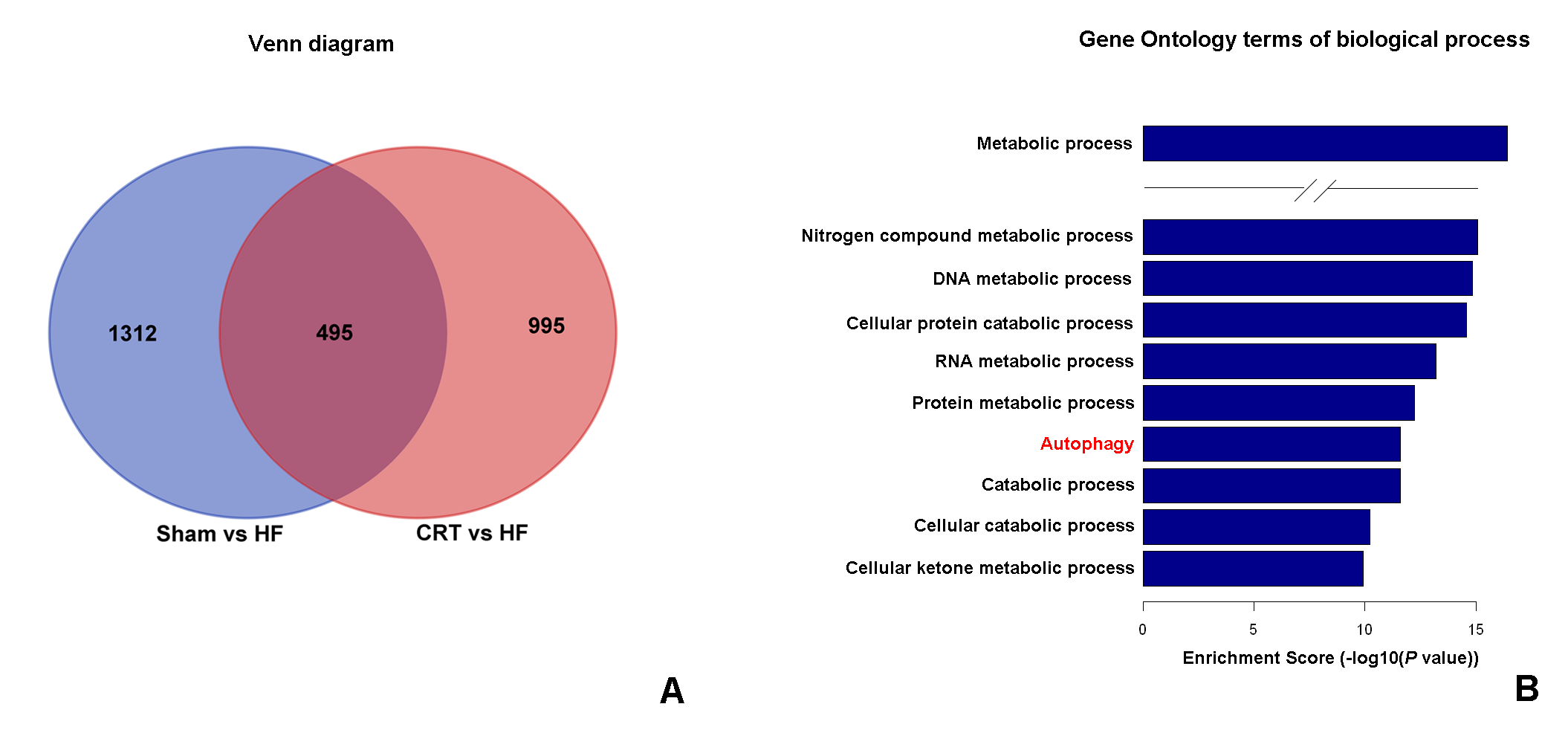
A: the overlapping area indicates the common genes differentially expressed in each data set (sham vs HF set and CRT vs HF set)

B: the enrichment analysis of differentially expressed genes based on GO terms indicates the autophagy is one of the most significant biological process in CRT treated heart failure.

**Supplemental figure 7. The protocol of the pathological condition induction and cell stretching pattern diagram**


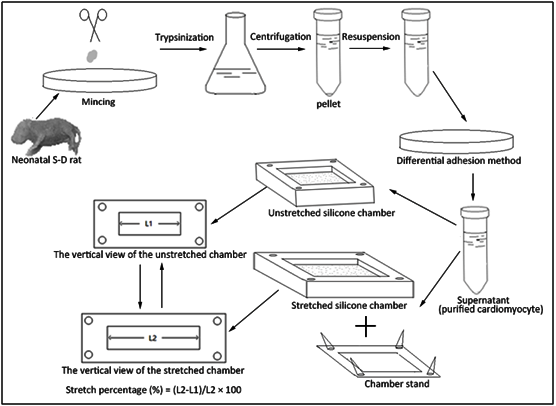
 **A**


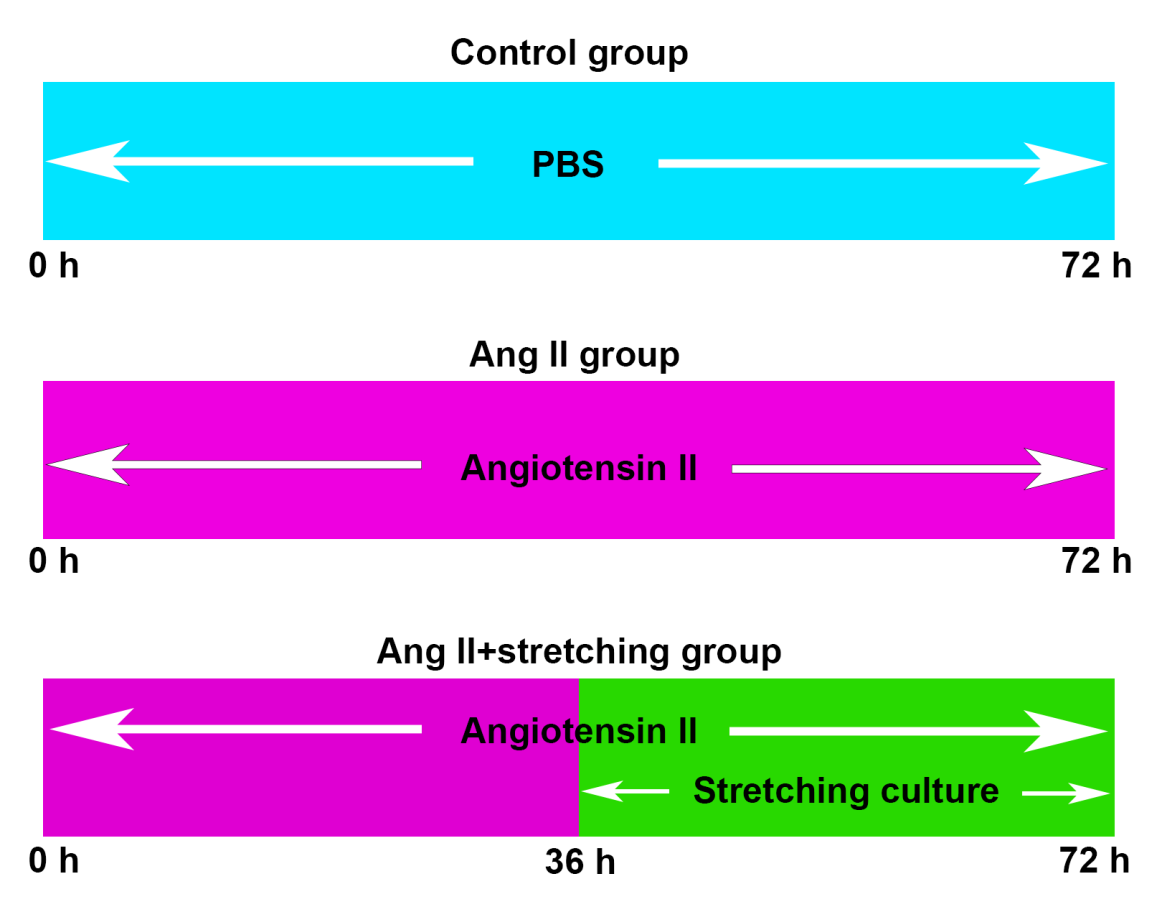
 **B**

This figure illustrates the key procedure of the cell stretching culture based on the process of the isolation of neonatal primary cardiomyocyte through differential adhesion method.

**Supplemental figure 8. The cellular mechanical stretching reversing Ang II induced cell enlargement**


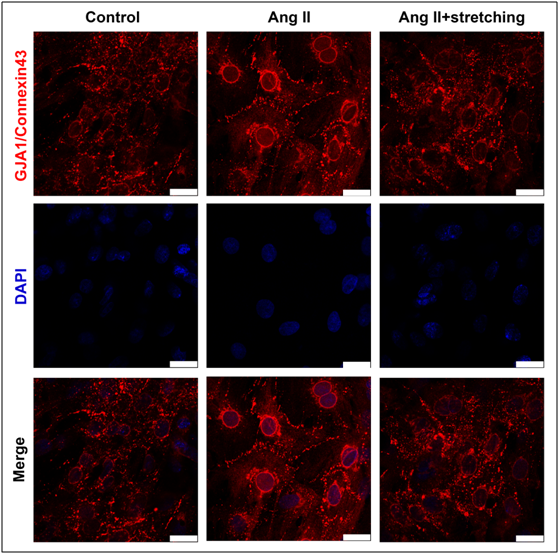


**A**

**
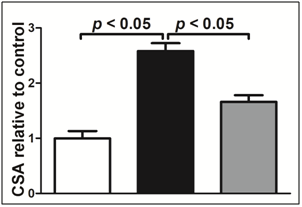
B**

The white scale bar referring to 25μm White column refers to sham group; black column refers HF group; grey column refers to HF + CRT group. CSA: cellular cross-sectional area

**Supplemental figure 9. The effects of small interfering RNA (siRNA) mediated Mcu knock-down on autophagy/mitophagy**


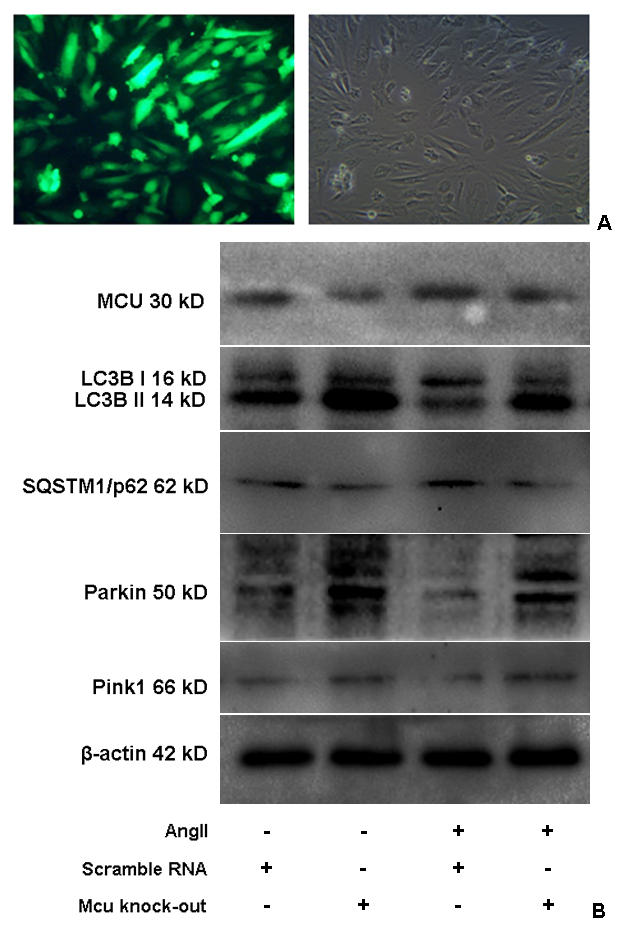


Mcu interfering sequence: GCTGACTGCCGGCTGCTTTCC; A: siRNA plasmid transfection efficiency indicates that the rate of GFP positive H9c2 is more than 80%; B: Mcu ablation is verified by western blotting. Ang II induced cell injury showed impaired autophagy and mitophagy in control cells, while Mcu knock-down could partly restore them. Therefore, intervention on Mcu could protect from pathological injury by autophagy/mitophagy enhancement.
